# End-to-end learning of 3D phase-only holograms for holographic display

**DOI:** 10.1038/s41377-022-00894-6

**Published:** 2022-08-03

**Authors:** Liang Shi, Beichen Li, Wojciech Matusik

**Affiliations:** Computer Science and Artificial Intelligence Laboratory, Massachusetts Institute of Technology, 32 Vassar St, Cambridge, MA 02139 USA

**Keywords:** Displays, Optical manipulation and tweezers

## Abstract

Computer-generated holography (CGH) provides volumetric control of coherent wavefront and is fundamental to applications such as volumetric 3D displays, lithography, neural photostimulation, and optical/acoustic trapping. Recently, deep learning-based methods emerged as promising computational paradigms for CGH synthesis that overcome the quality-runtime tradeoff in conventional simulation/optimization-based methods. Yet, the quality of the predicted hologram is intrinsically bounded by the dataset’s quality. Here we introduce a new hologram dataset, MIT-CGH-4K-V2, that uses a layered depth image as a data-efficient volumetric 3D input and a two-stage supervised+unsupervised training protocol for direct synthesis of high-quality 3D phase-only holograms. The proposed system also corrects vision aberration, allowing customization for end-users. We experimentally show photorealistic 3D holographic projections and discuss relevant spatial light modulator calibration procedures. Our method runs in real-time on a consumer GPU and 5 FPS on an iPhone 13 Pro, promising drastically enhanced performance for the applications above.

## Abstract (web summary)

Combining supervised and unsupervised learning, a new machine-learning system synthesizes high-quality 3D phase-only holograms end-to-end without human intervention and corrects vision aberrations.

## Graphical abstract

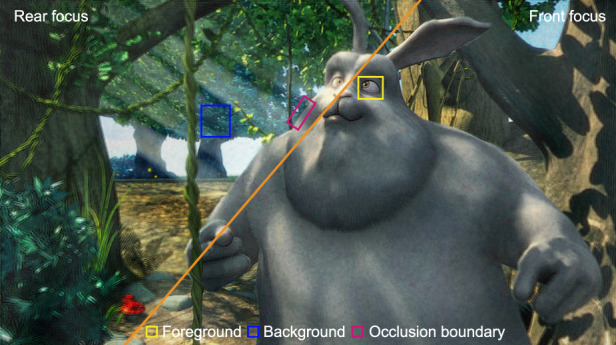

## Introduction

Computer-generated holography (CGH) is the method of digitally generating holographic interference patterns^1^. The interference patterns form a hologram that diffracts the incident light and establishes 3D images in the free space. This volumetric beam-shaping capability is critical to applications such as neural photostimulation^2,3^, optical/acoustic trapping^4,5^, and 3D displays^6,7^. While the first two applications often merely require focusing tens or hundreds of sparsely scattered 3D dots simultaneously, this number grows to millions for the display application. This imposes significant algorithmic and computational challenges in creating holographic video systems. Besides, existing spatial light modulators add another layer of complicacy by requiring an amplitude-only or a phase-only hologram^8^.

Both challenges are traditionally tackled by physical simulation with direct encoding or iterative optimization. Simulation-based methods represent the scene in point clouds^9^, light fields^10,11^, polygon meshes^12,13^, an RGB-D image^14^, or a multi-layer image^14,15^, and numerically simulate diffraction and interference using the angular spectrum method^16^ or Kirchhoff/Fresnel diffraction. The resulting complex hologram is directly converted to a phase-only hologram via amplitude discarding, temporal averaging, or the double phase method (and its variants)^6,14,17,18^. These methods work for continuous 3D scenes; however, the simulation step is typically time-consuming and the encoding step either works unreliably or requires manual tuning to find the optimal filtering parameters to achieve artifact-free results with minimal artificial blur. Alternatively, optimization-based methods use phase retrieval techniques^19^ or (stochastic) gradient descent^20,21^ to iteratively find a phase-only pattern whose propagated wavefront follows the target amplitude distribution. While similarly time-consuming, these methods may automatically discover phase-only solutions superior to the simulation-based methods and can be flexibly modeled for other downstream tasks such as static pupil expansion^22^, aberration correction^23,24^, contrast enhancement^25^, and bridging discrepancy between simulation and experiment^26^. Nevertheless, high-quality results were only demonstrated for 2D and multi-plane scenes^27^ instead of continuous 3D scenes due to the high computational cost that scales linearly with the depth resolution and the difficulty of specifying defocus responses at depth-varying regions and occlusion boundaries.

Recently, the differentiable nature of wave propagation and the maturity of differentiable software infrastructures has nurtured learning-based CGH algorithms that operate and improve upon the previous two methods to address the high computational cost. In particular, Deep-learning-generated holography (DGH)^28^ and Tensor Holography (TensorHolo)^6^ use simulation-based methods to synthesize a hologram dataset and employ supervised learning to train a convolutional neural network (CNN) as an efficient neural proxy of the simulator. Conversely, 3D-DGH^29^, DeepCGH^3^, and Neural Holography^24,27^ leverage unsupervised training by only specifying the desired amplitude at one or multiple depth planes and rely on the CNN itself to discover the optimal phase-only holograms analogous to the optimization-based methods. These networks significantly speed up the runtime, but they inherit the remaining problems associated with their parent methods. The employed 3D scene representations also have intrinsic limitations in depicting the complete scene geometry or providing high depth resolutions due to the necessity of CNN compatibility. Specifically, TensorHolo uses an RGB-D image, which only records the frontmost surface points. The lack of wavefront diffracted from the occluded points causes visible dark seams or brightness attenuation at the background side of the occlusion boundaries. In contrast, 3D-DGH and DeepCGH use a voxel grid represented as a multi-channel RGB image to accommodate occluded points; however, it becomes extremely memory-inefficient and computational-heavy when an ultra-high depth resolution, effectively a continuous 3D volume, is desired (i.e., hundreds or thousands of unique depth planes).

In this work, we propose a series of techniques to resolve the challenges above. Our techniques include the first use of a layered depth image^30^ as a data-efficient 3D representation for hologram synthesis, a new hologram dataset computed by the silhouette-mask layer-based method^15^ with ultra-dense layer partition (10,000 layers) to remove the remaining occlusion artifacts, a two-stage supervised+unsupervised training protocol that combines the benefit of both simulation-based and optimization-based methods, and a method to incorporate aberration correction into the 3D hologram learning pipeline. The resulting system, which we dubbed Tensor Holography V2, can directly synthesize photorealistic 3D phase-only holograms end-to-end without manual parameter tuning. It is robust to different image statistics, depth misalignment in real-world captured inputs, and different distance configurations between the hologram plane and the 3D volume. Besides, it can be customized for end-users with different vision aberrations. We experimentally demonstrate high image quality holographic 3D projection and aberration correction results. We also discuss the novel SLM calibration procedures used to achieve the demonstrated results. Table 1 lists the acronyms frequently used in this paper.

**Table 1:** Acronyms used in this paper

Acronyms	Phrase
LDI	Layered depth images^30^
SM-LBM	silhouette-mask layer-based method^15^
AA-DPM	anti-aliasing double phase method^6^
BL-DPM	band-limiting double phase method^18^
DDPM	deep double phase method
ASM	angular spectrum method^16^
TensorHolo	Tensor Holography^6^

## Results

### Layered depth images and silhouette-mask layer-based method

Occlusion modeling for 3D holograms is critical to the perceived realism. This task has been approached at the scene representation stage by rasterizing or ray-tracing an RGB-D image (with a depth map) to only record the frontmost visible surface^14,31^; rendering light fields to further account for view-dependent effects^11,32^. It has also been approached at the hologram computation stage via ray casting for point-based methods^33^ and silhouettes for FFT-based methods^12,34^. Both approaches are combined to prune the computation of non-line-of-sight objects and remove the wavefront of visible background objects occluded by the foreground^6^. In “Methods”, we examine the effectiveness of each approach and conclude that although more time-consuming, performing occlusion detection on the complete scene geometry is necessary to produce a physically correct response. Common representations that fully or largely serve this need include: (1) general point clouds; (2) meshes; (3) voxel grids; (4) light fields. Among them, general point clouds and meshes are CNN-unfriendly due to their scene-dependent feature lengths. Voxel grids operate in an explicit trade-off: the memory cost increases linearly as the depth resolution increases. Thus, it does not scale with high-resolution input, and the CNN’s first convolution layer bottlenecks the performance due to a vast number of input channels. For light fields, if pixels are treated as points^11,35^, it is inefficient as one point can be recorded multiple times in different views, and avoiding double-counting requires duplication detection; alternatively, if pixels are treated as rays^32^, generating high-quality sub-holograms will require an extremely dense angular sampling rate.

**Fig. 1:**
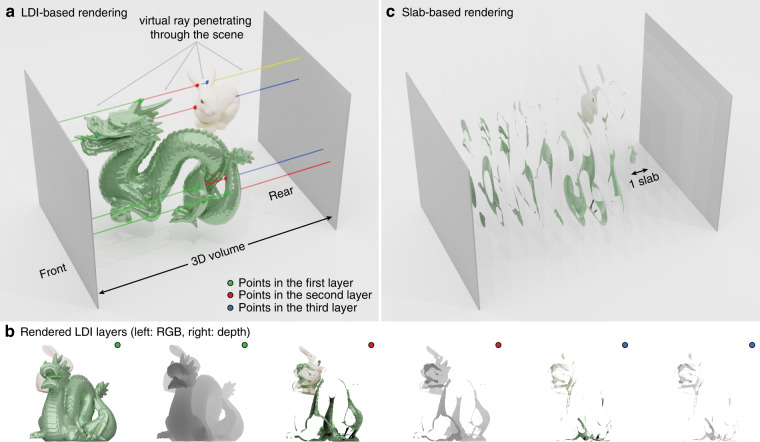
Comparison of LDI and voxel grid as 3D scene representation. **a** Rendering of an LDI records the intersections between a penetrating ray (colored differently after every new hit) and the scene objects (front-facing surfaces) at every spatial location. **b** First three layers of the LDI rendered for the scene in **a**. The number of valid pixels rapidly decreases as the index of the layer increases. **c** Rendering of a voxel grid that partitions the 3D scene into equal-length slabs and generates one RGB image for each slab. The depth resolution is proportional to the number of slabs

Here, we advocate a layered depth image (LDI) as an efficient 3D scene representation. LDI is a classical image-based rendering technique originally developed for novel view synthesis^30^. Unlike an RGB-D image that stores a single pixel at each spatial coordinate, it stores a sequence of RGB-D images along the line of sight originating from each spatial coordinate. Its first pixel records the first front-facing surface intersecting with the line of sight, and the second pixel records the second intersection assuming the line of sight pierces through the scene, and so forth till a maximum hit (layer) count is met (see Fig. 1a for an illustration). LDI has several unique advantages for holographic rendering applications. First, it is highly configurable such that if only a single layer is rendered, an LDI is equivalent to an RGB-D image; if all layers are exhaustively rendered, it losslessly encodes the entire scene for computing a physically correct hologram. In either case or for a limited number of layers, any point in the scene is only recorded once or discarded. Second, unlike a voxel grid, an LDI records the exact depth for every hit, decoupling the depth resolution with the number of LDI layers. Third, the sequence of pixels is recorded in a depth-sorted manner with wavefront from further layers providing a diminishing contribution to the hologram due to the occlusion of the frontal layers. Consequently, we find that a few LDI layers (i.e., 5) are sufficient to produce a close-to-ground-truth hologram, and thus it is highly data-efficient. Fourth, once the number of layers is set, its feature length is fixed and independent of scene complexity; thus, it is CNN-friendly. Finally, it can be efficiently rendered by the existing real-time graphics pipeline via depth peeling (see “Methods” for details).

To compute a 3D hologram from an LDI, ray casting can be performed from each point’s belonging mesh at the recorded 3D location. However, geometric occlusion for diffraction calculation has been shown to cause minor artifacts. Because runtime is ultimately unimportant for dataset synthesis, we propose using the silhouette-mask layer-based method^15^ (SM-LBM) with ultra-dense depth partition to avoid the mixed-use of geometric and wave optics models. SM-LBM was originally proposed to receive a voxel grid input generated by slab-based rendering (see Fig. 1b), which does not scale with increasing depth resolution. Using SM-LBM with LDI is straightforward. Any non-zero pixel in an LDI defines a valid 3D point before depth quantization. When the number of depth layers *N* is determined, each point is projected to its nearest plane, and a silhouette is set at the same spatial location. Denote the complex amplitude distribution of the *N*th layer $L_N \in {\Bbb C}^{R_x \times R_y}$ as

1 $$L_N = A_N{{{\mathrm{exp}}}}\left( {{\rm{i}}\frac{{2\pi z_N}}{\lambda }} \right)$$

here, *z*_*N*_ is the signed distance from the *N*th layer to the hologram plane, where a negative distance denotes a layer behind the hologram plane and vice versa, $A_N \in {\Bbb R}^{R_x \times R_y}$ is the amplitude of the layer, *R*_*x*_, and *R*_*y*_ are the spatial resolution along the *x* and *y* axis. The exponential term defines the layer’s initial phase following Maimone et al.’s formula to cause a smooth and roughly zero-mean phase profile at the hologram plane^14^. We use the angular spectrum method to propagate *L*_*N*_ to the location of *N*−1th layer

2 $$\begin{array}{*{20}{l}} {C_{N - 1} = {{{\mathrm{ASM}}}}\left( {L_N,d_l} \right)} \\\qquad\quad { = {{{\mathcal{F}}}}^{ - 1}\left( {{{{\mathcal{F}}}}\left( {L_N} \right) \odot \exp \left( {{\rm{i}}2\pi d_l\sqrt {\lambda ^{ - 2} - \mu ^2 - v^2} } \right)} \right)} \end{array}$$

where $\mu \in {\Bbb R}^{R_x \times R_y}$ and $\upsilon \in {\Bbb R}^{R_x \times R_y}$ are the spatial frequencies along the *x* and *y* directions, *d*_*l*_ is the layer thickness (positive), $\odot$ denotes Hadamard element-wise product, ${{{\mathcal{F}}}}$ and ${{{\mathcal{F}}}}^{ - 1}$ are the 2D Fourier transform and inverse Fourier transform operator. *C*_*N*–1_ is multiplied by the binary silhouette mask at the *N*−1 layer

3 $$M_{N - 1}\left( {x,y} \right) = \left\{ {\begin{array}{*{20}{l}} {0,\;\;A_{N - 1}(x,y) \,>\, 0} \\ {1,\;\;A_{N - 1}(x,y) = 0} \end{array}} \right.$$

and the complex amplitude at the *N*−1 layer is updated by adding the masked complex field

4 $$L_{N - 1} = C_{N - 1}M_{N - 1} + L_{N - 1}$$

Iterating this process until reaching the first layer, the final complex hologram is obtained by propagating the updated first layer to the hologram plane.

We further augment SM-LBM with aberration correction at the cost of computational efficiency. Reconsidering the forward propagation of *N*th layer *L*_*N*_, we only process the occlusion of the frontal layer without adding their content, namely removing the second addition term in Eq. (4). After processing the occlusion of all frontal layers, we propagate the resulted wavefront back to the starting location of *L*_*N*_ to obtain an occlusion-processed *L*′_*N*_. We then perform aberration correction in the frequency domain

5 $$L^\prime_{N_C} = {{{\mathcal{F}}}}^{ - 1}\left( {{{{\mathcal{F}}}}(L^\prime _N) \odot \Phi _{z_N}} \right)$$

where $\Phi _{z_N} \in {\mathbb{C}}^{R_x \times R_y}$ is a depth-dependent global aberration correction kernel in the Fourier domain. We detail the process to obtain $\Phi_{z_{N}}$, and an extension to model depth-dependent spatially-varying aberration correction in “Methods”. Finally, $L^{\prime}_{\,N_C}$ is propagated to the target hologram plane. This procedure is repeated independently for the content at every discretized depth (i.e., from layer 1 to *N*) and integrating the results of all procedures at the target plane forms the final hologram. Note that the required total number of propagation operations increases to *N*^2^/2 compared with *N* in the standard SM-LBM. This prevents *N* from being extremely high if aberration correction is needed, but the computational resource is limited.

### MIT-CGH-4K-V2

SM-LBM and its aberration-correction variant are slow due to sequential occlusion processing. To improve the performance, we generate a new dataset with LDIs and SM-LBM holograms and train a CNN to accelerate inference. Generating this dataset requires setting three critical parameters: the depth of the 3D volume, the number of layers used by LDIs, and the number of layers (depth resolution) used by SM-LBM.

**Fig. 2:**
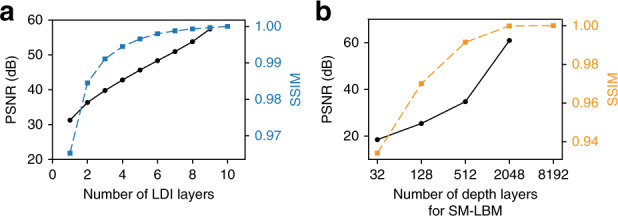
SM-LBM hologram quality as a function of LDI layers and depth discretization. **a** The addition of more LDI layers provides diminishing returns on the hologram fidelity and the improvement plateaus after 5 layers. **b** A depth resolution over 11 bits (2048) results in a negligible difference in the image quality

We set the 3D volume depth to be 6 mm under collimated illumination to facilitate quantitative comparison with the publicly available TensorHolo V1 network^6^, and similarly for the random scene configuration. To determine the number of layers for LDIs, we compute the mean peak signal to noise ratio (PSNR) and the mean structure similarity index^36^ (SSIM) for the amplitude maps of the holograms computed from LDIs with $N = 1,2, \cdots ,9$ layers against the ones computed from LDIs with *N* = 10 layers (after which we observe few valid pixels) over 10 random scenes. The mean SSIM plateaus after *N* = 5 (see Fig. 2a), reflecting a diminishing improvement with more layers. Thus, we choose *N* = 5 for this work, but more layers can be used for higher accuracy. Similarly, to determine the number of layers for SM-LBM, we compute the holograms using $2^{N_d}$ layers for *N*_*d*_ = 5, 7, 9, and 11, and compare the mean PSNR and the mean SSIM of these holograms against the ones computed from *N*_*d*_ = 13 over 10 random scenes. The mean SSIM plateaus after *N*_*d*_ = 11 (see Fig. 2b), indicating negligible improvement for the 3D image quality. Nevertheless, we use a partition of 10,000 layers (13.28-bit depth) as a showcase, which has not been demonstrated previously. We rendered MIT-CGH-4K-V2, a new hologram dataset with 4000 pairs of LDIs and holograms with 3800 for training, 100 for validation, and 100 for testing at 384 × 384 pixels similar to TensorHolo V1.

### End-to-end learning of 3D phase-only hologram

In TensorHolo V1, the CNN directly maps the input RGB-D image to the midpoint hologram, a wavefront recording plane set to the center of the 3D volume that minimizes the sub-hologram width, and phase-only encoding is performed separately using anti-aliasing double phase encoding (AA-DPM). However, both AA-DPM and an analogous band-limiting double phase method (BL-DPM)^18^ require manual tuning to find the optimal low-pass filtering parameters that produce an artifacts-free image with the minimal artificial blur. For video or live streaming inputs where manual tuning is infeasible, highly conservative filtering is required to keep the entire sequence artifacts-free. Meanwhile, the low-pass filterings in both methods are either spatially-invariant or frequency-invariant; thus they do not adapt to the content and may incur unnecessary filtering. In addition, we observe the minimal filtering strength of both methods grows with the sub-hologram width, leading to degraded performance when the 3D frustum is placed far against the hologram plane. In practice, this limits the image quality when designing compact holographic near-eye displays, where it is ideal for reducing the physical distance between the SLM and the eyepiece by computationally shifting the 3D image tens of millimeters behind the hologram.

Although a CNN can be trained to directly predict an unconstrained 3D phase-only hologram using unsupervised learning by only forcing the focal stack to match the one produced by the target complex hologram^3^, ablation studies have shown that removing the supervision of ground truth complex hologram noticeably degrades the image quality^6^ and enforcing the phase-only constraint can only worsen the performance. Moreover, direct synthesis of phase-only holograms prevents using midpoint holograms to reduce computational costs since learning an unconstrained midpoint phase-only hologram does not guarantee a uniform amplitude at the target hologram plane.

**Fig. 3:**
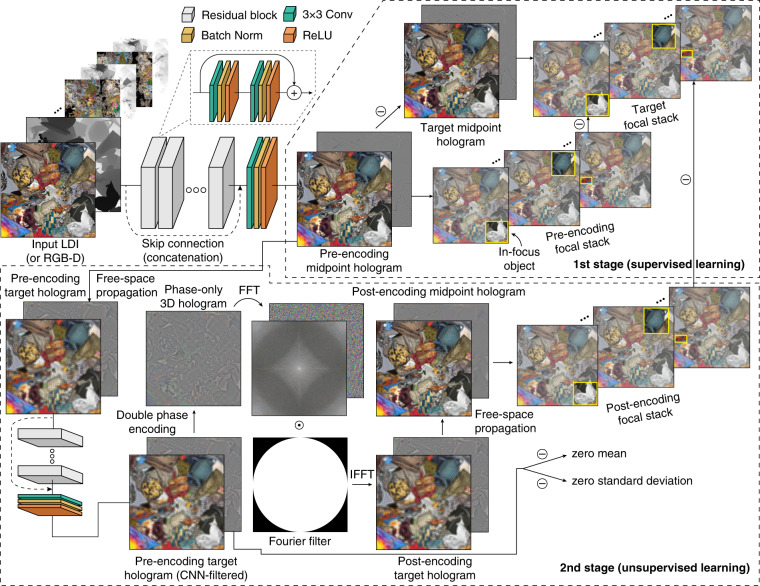
Overview of the training procedure. In the first stage, the CNN is trained to reproduce the ground truth midpoint hologram with direct supervision and a dynamic focal stack loss. In the second stage, the CNN prediction is propagated to the target hologram plane, filtered by a second CNN, double phase encoded, Fourier filtered, and back propagated to the center of the 3D volume to obtain the post-encoding midpoint hologram. No ground truth phase-only hologram is provided, and the CNN is trained to discover an optimal solution with the dynamic focal stack loss between the post-encoding focal stack and the target focal stack plus a regularization loss (see Eq. (11)). The Fourier space amplitude is visualized in the logarithmic scale

We propose a two-stage supervised+unsupervised training to overcome these challenges (see Fig. 3 for visualization). The key insight is to keep using the double phase principle to perform phase-only encoding for retaining the advantage of learning the midpoint hologram while embedding the encoding process into the end-to-end training pipeline and relegating the CNNs to discover the optimal pre-encoding complex hologram through unsupervised training. We detail the training process below and refer to this neural phase-only conversion method as the deep double phase method (DDPM).

The first stage supervised training is identical to TensorHolo V1 despite that we train two versions of CNNs in V2. Both are trained to predict the target midpoint hologram computed from the LDI input, but one receives the full LDI, and the other receives only the first layer of the LDI, the same RGB-D input as the V1 network receives. The latter CNN has an additional job of inferring the occluded points close to the depth boundaries and filling in their missing wavefront. It is particularly useful for reducing the rendering overhead and reproducing real-world scenes captured as RGB-D images, where physically capturing a pixel-aligned LDI is nearly impossible.

Once the CNN excels at this task, we initialize the second stage unsupervised training by applying a chain of operations to the network-predicted midpoint hologram $\tilde H_{{{{\mathrm{mid}}}}} = \tilde A_{{{{\mathrm{mid}}}}}{\rm{e}}^{{\rm{i}}\tilde \phi _{{{{\mathrm{mid}}}}}}$. First, it is propagated to the target hologram plane and pre-processed by a second CNN to yield the pre-encoding target hologram prediction

6 $$\begin{array}{lll} \tilde H_{{{{\mathrm{tgt}}{\mbox{-}}{\rm{pre}}}}}&= &\tilde a_{{{{\mathrm{tgt}}{\mbox{-}}{\rm{pre}}}}}\tilde A_{{{{\mathrm{tgt}}{\mbox{-}}{\rm{pre}}}}}{\rm{e}}^{{\rm{i}}\tilde \phi _{{{{\mathrm{tgt}}{\mbox{-}}{\rm{pre}}}}}} \\&=& {{{\mathrm{CNN}}}}_{{{{\mathrm{filter}}}}}\left( {{{\mathrm{ASM}}}}\left( {\tilde H_{{{{\mathrm{mid}}}}},d_{{{{\mathrm{offset}}}}}} \right){{{\mathrm{exp}}}}\left( {{\rm{i}}\frac{{2\pi d_{{{{\mathrm{offset}}}}}}}{\lambda }} \right) \right) \end{array}$$

where *d*_offset_ is the signed distance from the midpoint hologram to the target hologram plane, $\tilde A_{{{{\mathrm{tgt}}{\mbox{-}}{\rm{pre}}}}}$ is the normalized amplitude, and $\tilde a_{{{{\mathrm{tgt}}{\mbox{-}}{\rm{pre}}}}}$ is the scale multiplier. The second CNN serves as a content-adaptive filter to replace the Gaussian blur in AA-DPM. The exponential phase correction term ensures that the phase after propagation is still roughly centered at 0 for all color channels. It is also critical to the success of AA-DPM, which minimizes phase wrapping. Next, the standard double phase encoding is applied to obtain a phase-only hologram

7 $$P\left( {x,y} \right) = \left\{ {\begin{array}{*{20}{l}} {0.5\tilde a_{{{{\mathrm{tgt}}{\mbox{-}}{\rm{pre}}}}}{\rm{e}}^{{\rm{i}}\left( {\tilde \phi _{{{{\mathrm{tgt}}{\mbox{-}}{\rm{pre}}}}}\left( {x,\,y} \right) \,-\, \cos ^{ - 1}\tilde A_{{{{\mathrm{tgt}}{\mbox{-}}{\rm{pre}}}}}\left( {x,\,y} \right)} \right)},\;\;x + y\;{{{\mathrm{is}}}}\;{{{\mathrm{odd}}}}} \\ {0.5\tilde a_{{{{\mathrm{tgt}}{\mbox{-}}{\rm{pre}}}}}{\rm{e}}^{{\rm{i}}\left( {\tilde \phi _{{{{\mathrm{tgt}} {\mbox{-}} {\rm{pre}}}}}\left( {x,\,y} \right)\, +\, \cos ^{ - 1}\tilde A_{{{{\mathrm{tgt}}{\mbox{-}}{\rm{pre}}}}}\left( {x,\,y} \right)} \right)},\;\;x + y\;{{{\mathrm{is}}}}\;{{{\mathrm{even}}}}} \end{array}} \right.$$

and no pre-blurring is applied in contrast to AA-DPM. Third, the phase-only hologram is filtered in the Fourier space to obtain the post-encoding target hologram prediction

8 $$\tilde H_{{{{\mathrm{tgt}}{\mbox{-}}{\rm{post}}}}} = {{{\mathcal{F}}}}^{ - 1}({{{\mathcal{F}}}}(P) \odot M_{{{{\mathrm{Fourier}}}}})$$

where *M*_Fourier_ models a circular aperture in the Fourier plane

9 $$M_{{{{\mathrm{Fourier}}}}}\left( {x,y} \right) = \left\{ {\begin{array}{*{20}{l}} {1,\;\;x^2 + y^2 \le r^2} \\ {0,\;\;x^2 + y^2 \,>\, r^2} \end{array}} \right.$$

Here, *r* is the radius of the aperture in the pixel space. We set it to half of the image resolution, letting the entire first-order diffraction pass through the physical aperture. Finally, the post-encoding target hologram prediction is propagated back to yield the post-encoding midpoint hologram

10 $$\tilde H_{{{{\mathrm{mid}}{\mbox{-}}{\rm{post}}}}} = {{{\mathrm{ASM}}}}(\tilde H_{{{{\mathrm{tgt}}{\mbox{-}}{\rm{post}}}}}, -\, d_{{{{\mathrm{offset}}}}})$$

By appending these operations, the second stage unsupervised training fine-tunes the CNN prediction using the dynamic focal stack loss calculated between the post-encoding midpoint hologram and the ground truth midpoint hologram, plus a regularization loss on the pre-encoding target hologram phase

11 $$l_{{{{\mathrm{tgt}}{\mbox{-}}{\rm{pre}}}}} = \sqrt {\frac{{{\sum} {\left( {\widetilde \phi _{{{{\mathrm{tgt}}{\mbox{-}}{\rm{pre}}}}} - \overline {\widetilde \phi _{{{{\mathrm{tgt}}{\mbox{-}}{\rm{pre}}}}}} } \right)^2} }}{{R_xR_y}}} + \overline {\widetilde \phi _{{{{\mathrm{tgt}}{\mbox{-}}{\rm{pre}}}}}}$$

where $\bar \cdot$ denotes the mean operation. The regularization loss encourages the pre-encoding target hologram phase to be zero mean and exhibit a small standard deviation. This term minimizes phase wrapping during the double phase encoding, which may not affect the simulated image quality but degrade the experimental result. Without this loss, the unregulated phase exhibits a large standard deviation and shifts away from zero mean, leading to non-negligible phase wrapping, especially when the maximum phase modulation is limited to 2*π*.

In the second training stage, direct supervision from the ground truth midpoint hologram is intentionally ablated. This expands the solution space by allowing the CNNs to freely explore the neural filtering to optimally match the ground truth focal stack, which a user ultimately sees. It also facilitates regularization on the pre-encoding target hologram phase to better handle hardware limitations (i.e., limited range of phase modulation). In practice, the resulting prediction of the post-midpoint hologram phase visually differs from the ground truth as high-frequency details are attenuated or altered in a spatially-varying and content-adaptive manner to avoid speckle noise. With direct supervision that encourages retention of high-frequency details, we find it negatively impacts speckle elimination.

Collectively, the proposed two-stage training first excels at reproducing the ground truth complex 3D holograms at all levels of detail, then fine-tunes a display-specific CNN for fully automatic speckle-free 3D phase-only hologram synthesis. The second training stage takes fewer iterations to converge; therefore, it is efficient to optimize multiple CNNs for different display configurations upon the completion of the first training stage. The training process is detailed in “Methods”.

### Simulation and experimental verification

**Fig. 4:**
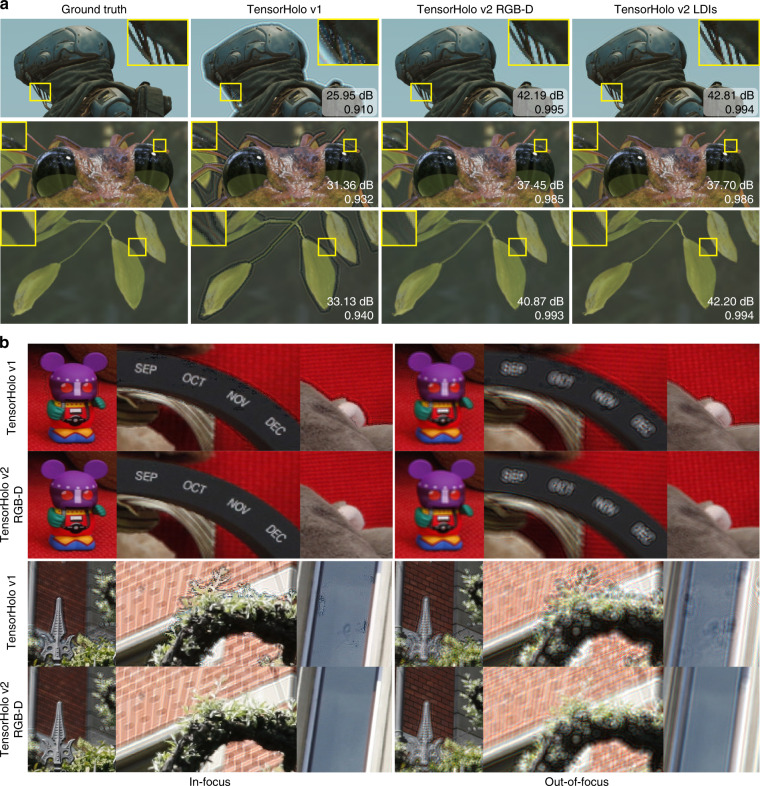
Performance evaluation of TensorHolo v1, TensorHolo v2-RGB-D, and TensorHolo v2-LDIs. **a** Comparison of 3D holograms for computer-rendered scenes. The yellow inset marks the region of interest. **b** Comparison of 3D holograms for real-world captured scenes

We qualitatively and quantitatively evaluate TensorHolo V2 CNNs in simulation. Figure 4 compares the depth-of-field images reconstructed by the complex hologram prediction of the V1 CNN, the V2 CNNs Stage 1, and the ground truth. The rendered LDIs and real-world captured RGB-D inputs can be found in “Methods”. The V2 RGBD-CNN largely removes the artifacts at the occlusion boundaries, compensates the missing wavefront with a plausible estimation of the occluded pixels, and is robust to depth misalignment in the real-world captured inputs (see Fig. 4b). When the foreground of the depth boundary has extraordinary pixels, such as the specular white dot in the yellow inset of the frog scene (see Fig. 4a, Row 2 Column 3), the V2 RGBD-CNN can mis-extrapolate the occlusion and produce an inaccurately defocused background. The V2 LDI-CNN eliminates this error with the LDI input (see Fig. 4a, Row 2 Column 4). On the challenging validation set, V2 RGBD-CNN and V2 LDI-CNN perform similarly, outperforming the V1 CNN and Maimone et al. by a large margin (see Table 2).

**Table 2:** Methods comparison on the validation set

Methods	PSNR (dB)	SSIM
Maimone et al.^14^	21.6 (21.3)	0.816 (0.808)
Shi et al.^6^	23.6 (23.2)	0.830 (0.821)
Ours (RGB-D)	29.4 (28.9)	0.945 (0.942)
Ours (LDI)	29.6 (29.1)	0.947 (0.944)

The unbracketed result is calculated for the amplitude map of the predicted hologram, the bracketed result is calculated by averaging the results of a focal stack with 15 most frequent depths and 5 random depths for each image.

**Fig. 5:**
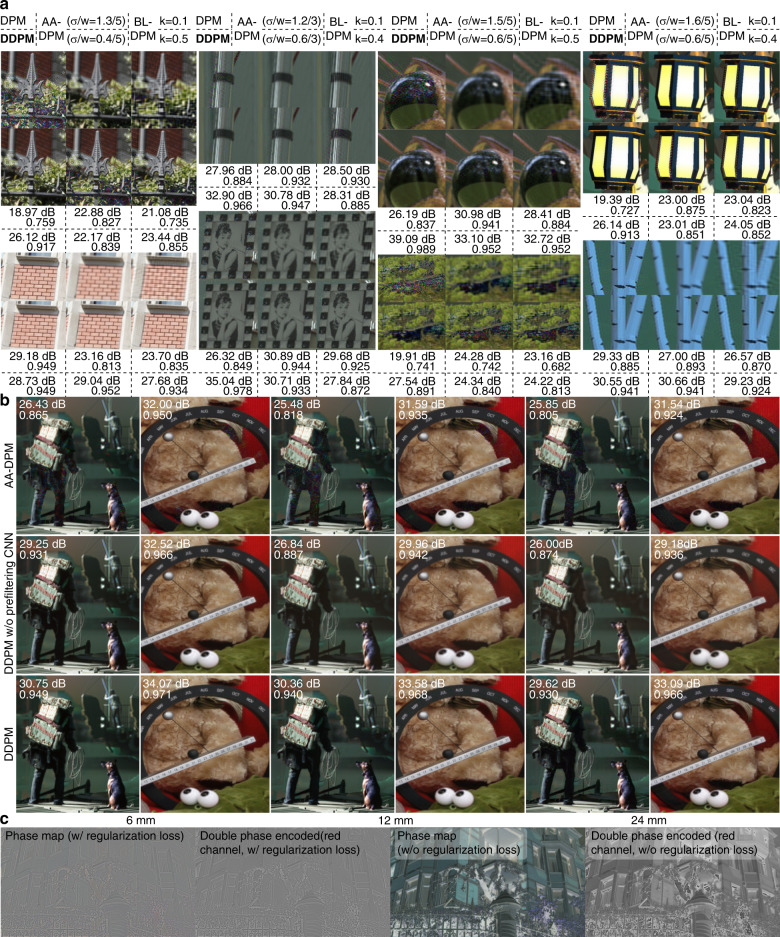
Performance evaluation of DDPM. **a** Comparison of simulated reconstruction using DDPM, AA-DPM, and BL-DPM for midpoint hologram. For AA-DPM and BL-DPM, two filter strengths are applied: the weaker one results in similar sharpness to DDPM; the stronger one results in an artifact-free image. **b** Comparison of simulated reconstruction using DDPM, DDPM (without the pre-filtering CNN), and AA-DPM for target holograms with 6, 12, and 24 mm offset to midpoint hologram. The AA-DPM filter strengths used for the boat and the couch scene are (σ = 1.0, *w* = 5) and (σ = 0.8, *w* = 3) respectively. They are the minimal strengths that result in artifact-free images for the midpoint hologram. **c** Comparison of pre-encoding target hologram phase and its double-phase encoded phase-only hologram predicted by CNNs trained with and without the regularization loss. The hologram plane is offset by 6 mm

DDPM robustly performs the phase-only conversion at detail-rich, high-frequency, high-contrast, and low-amplitude regions with full automation (see Fig. 5a and Supplementary Video 1/2). In all examples, DDPM simultaneously achieves high resolution and speckle-free reconstruction. In contrast, AA-DPM and BL-DPM have to trade between resolution and speckle noise via different filter strengths. Benefiting from the display-specific training, DDPM maintains the image resolution when hologram planes are offset against the 3D volume (see Fig. 5b, bottom), and the addition of the pre-filtering CNN preserves the image contrast (see the human limbs and perpetual calendar in Fig. 5b, middle). In opposite, using AA-DPM with filter strength optimized for the midpoint hologram is inadequate and produces ringing artifacts at distant hologram planes (see Fig. 5b, top). Successful DDPM training benefits from the regularization loss to minimize phase wrapping (see Fig. 5c).

**Fig. 6:**
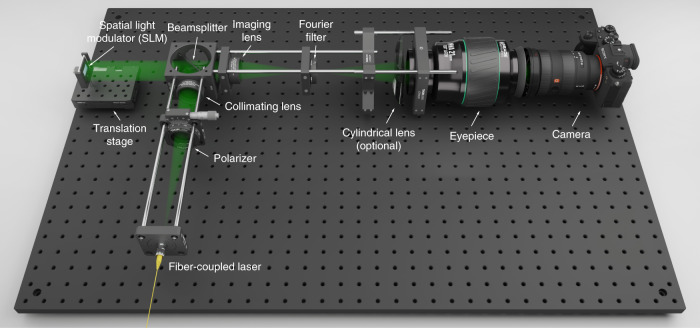
Schematic of the experimental holographic display prototype. The cylindrical lens is used for the aberration-correction experiment (see Fig. 8). The control box of the laser and Labjack DAQ are not visualized

**Fig. 7:**
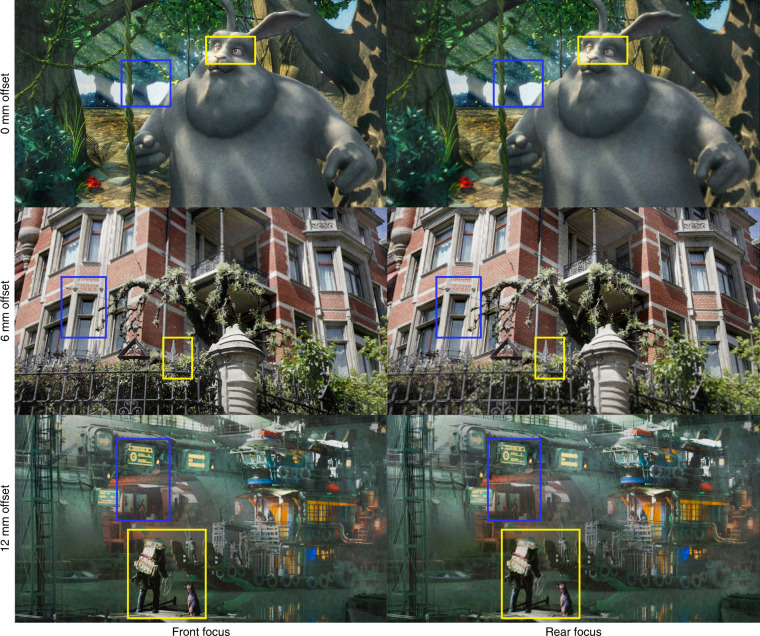
Experimental demonstration of holographic 3D projections. Two computer-rendered (Top/Bottom) and one real-world captured (Middle) scenes are displayed and photographed for target holograms located at three different distances (marked on the left) to the midpoint hologram. The yellow box marks the foreground object of interest, and the blue box marks the background object of interest. Readers are encouraged to zoom in to examine the image details

A benchtop holographic display is constructed and calibrated for experimental verification (see Fig. 6 for a schematic visualization and see Methods for hardware and calibration details). We photographed 3D holograms of rendered and real-world scenes generated by the V2 RGBD-CNN (see Fig. 7). The hologram planes are offset differently from the 3D scenes to evaluate the DDPM performance.

Compared to supplementary video 4 of TensorHolo V1, the Big Buck Bunny result (see Fig. 7 top and Supplementary Video 3 for continuous 3D refocusing) no longer present visible dark seams around foreground objects such as the bunny body and tree vines. The mansion result (Fig. 7, middle) validates TensorHolo V2’s robustness to depth misalignment. Unlike Extended Data Fig. 9 of TensorHolo V1, artifacts on the blue window, fence, and shrubs are eliminated, and the resulting 3D image is more naturally defocused. Reconstructed at a further distance, the boat result (Fig. 7, bottom) reproduces smooth color gradients of the wall and the water surface owing to accurate phase control from a per-pixel voltage-to-phase look-up table (see Methods for details). The human and dog in the foreground are absent from the speckle noise that would otherwise appear if AA-DPM was used (see Fig. 5b). In all results, the V2 RGBD-CNN consistently produces realistic depth boundaries using a single RGB-D input. Supplementary Video 4 demonstrates real-time computation and playback of 3D holograms calculated for cellphone-captured RGB-D videos, where the V2 RGBD-CNN robustly handles depth misalignment.

**Fig. 8:**
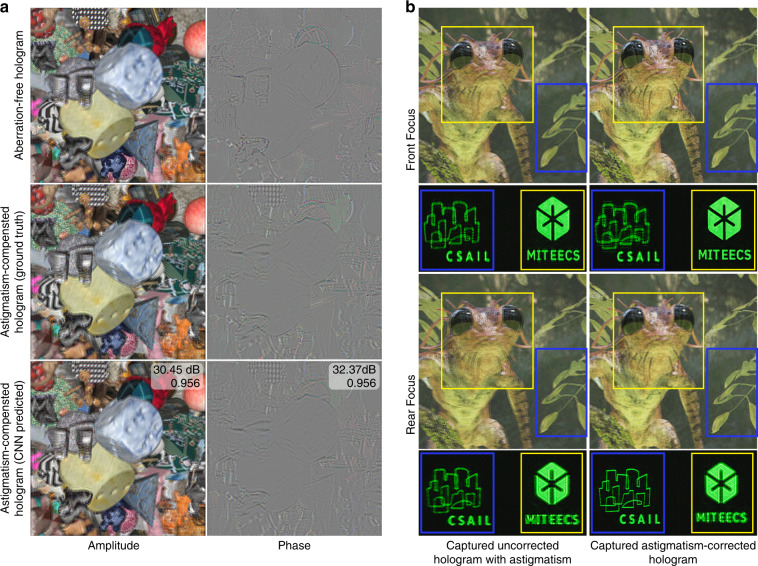
Performance evaluation of vision aberration correction. **a** Top: A hologram calculated for normal vision. Middle: A hologram calculated using the proposed variant of SM-LBM that corrects a synthetic astigmatic vision induced by a 200 mm cylindrical lens. The calibration method is detailed in Methods. Bottom: The CNN trained on the astigmatism-corrected dataset jointly performs diffraction simulation and aberration correction. The PSNR and SSIM of the predicted hologram are marked on the top right corner. **b** Experimentally captured CNN-predicted holograms of a natural scene and a test scene without (Left) and with (Right) astigmatism correction. The yellow and the blue box marks the foreground and the background object of interest, respectively. The in-focus object in the corrected hologram exhibits horizontal blurs in the uncorrected hologram (i.e., treefrog’s tentacles, eye reflections, tree leaves, and test patterns)

To demonstrate aberration correction, we place a cylindrical lens with a focal length of 200 mm in front of the eyepiece to cause astigmatism (see Fig. 6). The cylinder axis is placed vertically, causing the originally in-focus object to primarily exhibiting horizontal blurs. We performed system identification (see Methods for details) and generated an aberration-corrected dataset with 256 (8-bit) depth levels. The trained network predicts holograms that compensate for aberration for the random scene in the validation set (see Fig. 8a), natural scenes, and test patterns (see Fig. 8b). The captured front focus image in the tree frog scene closely resembles the simulation of an unaberrated system in Fig. 5a.

## Discussion

Holographic 3D displays provide differentiating interactive experiences from cell phones or stereoscopic augmented reality (AR) and virtual reality (VR) displays. TensorHolo V2 makes a step towards the end-to-end synthesis of 3D phase-only holograms. It is fully automatic, robust to rendered and misaligned real-world inputs, produces realistic depth boundaries, and corrects vision aberrations. Reusing the minimalistic CNN architecture in TensorHolo V1, it runs in real-time on a consumer-grade GPU and 5 FPS on an iPhone 13 Pro (see “Methods” for runtime performance details), promising real-time mobile performance for future-generation AR/VR headsets and glasses.

To produce a more visually engaging 3D experience and further reduce the computational complexity, many extensions to the current method are worth investigating. One is foveation-guided holographic rendering^37–40^. For stereoscopic and light-field AR/VR displays, foveated rendering lowers the image quality in the peripheral vision to reduce rendering cost^41–43^. In the context of holographic rendering, a 2D hologram with synthetic blur can be used for peripheral vision instead of a true 3D hologram. As eye trackers become widely available in next-generation head-mounted displays, this can be a powerful computation-only approach to improve the rendering performance.

Another direction is to support view-dependent effects. Although an LDI provides sufficient scene information from one perspective, view-dependent effects are not explicitly modeled since disoccluded regions or out-of-view objects will become visible from other views, as well as occlusion of currently visible points. However, the current localized 3D experience may be sufficient for head-mounted displays since holograms can be dynamically updated to reflect the changed user’s viewpoint as the rendering of LDI is efficient. Yet, a hologram that supports a view-dependent effect is beneficial when fabricating ultra-high-resolution static copies. To design a scene representation sufficient for modeling view-dependent effect, one could render multiple LDIs from evenly sampled viewpoints within an arc or a line of view space, and compact LDIs into a master LDI plus side information of disocclusion, new occlusion, and out of view scene through point cloud duplication detection. One could also replace LDI with recently emerged neural scene representation (i.e., NeRF^44^), which uses a coordinate-based neural network to memorize the entire 3D volume compactly. For hologram rendering, Zhang et al.^45^ showed that Fourier domain segmentation and stitching of multiple holograms rendered from different viewports could provide a continuous view-dependent effect for large baseline movement. Co-designing a learning-based system to incorporate both new input representations and new rendering schemes can further unleash the power of holograms when higher resolution SLMs become available.

The current system also requires a Fourier filter to shield higher-order diffractions for producing a clean image. Changing the image formation model to model higher-order diffractions explicitly can potentially remove the need for optical filtering^46^ and increase the method compatibility to enable more flexible display designs. Meanwhile, the current ground truth focal stack is rendered under the assumption of a coherent imaging model, the real-world depth of field yet follows the incoherent imaging model. This can create a mismatch of defocus blur when overlaying virtual objects with real-world scenes. Using an incoherent rendering model to generate the target focal stack while matching it under the constraint of the coherent propagation model may offer a more realistic 3D perception without breaking holography’s principle of operation. This can be further combined with temporal time-multiplexing to improve the image quality^47^.

The current system uses Maimone et al.’s phase initialization to suppress speckle noise; however, the artificially-enforced smooth phase distribution could computationally constrain the angular spectrum and lead to reduced eyebox. A potential way to maximize the eyebox is to add pupil modeling in the Fourier plane during the unsupervised training stage. Because the pre-encoding phase is not forced to match the target hologram, the filtering of eccentric pupils can encourage a broad angular spectrum bandwidth to maintain image quality for different pupil locations while keeping the speckle low.

When miniaturizing a holographic display into an eyeglass-like form factor, one inevitably deals with spatially-varying optical aberrations^14^. A successful demonstration of a learning system that produces aberration-corrected content for an eyeglass-like prototype will be necessary for industry adoption of holography.

Finally, the proposed system provides a limited immersive 3D experience due to the SLM’s low space-bandwidth product. Consequently, the development of SLMs with higher pixel density, faster refresh rate, higher bit depths, and possibly joint amplitude and phase modulation are imperative. Designing compact combining optics (i.e., holographic optical element (HoE) lens, geometric phase lens, and waveguides) with less aberration/more substantial optical power and novel optical path folding structures are also critical to the success of miniaturizing the display form factor^48,49^.

## Materials and methods

### Methods comparison for occlusion modeling

**Fig. 9:**
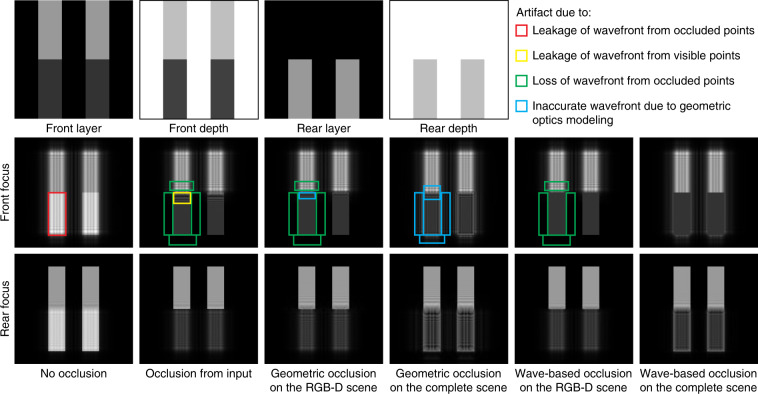
Methods comparison for occlusion modeling. The top 4 images constitute a 2-layer LDI that completely depicts the test scene. Different types of artifacts are only marked for the front focus images

We examine methods for occlusion modeling using a simple test scene consisting of two vertical bars with the front bar perfectly occluding the lower half of the rear bar (see Fig. 9, top). We assign different amplitudes to the front and rear bars and repeat the bar pair horizontally for visualization. The complete scene is represented by an LDI with two layers: the first layer for the front bar plus the top half of the rear bar and the second layer for the bottom half of the rear bar. The rasterized scene is represented by the LDI’s first layer. We tested six pairs of input and CGH algorithms:

1. No occlusion: apply the point-based method to the complete scene.
2. Occlusion only from input: apply the point-based method to the rasterized scene.
3. Geometric occlusion detection (input+computation): apply the occlusion-aware point-based method to the rasterized scene.
4. Geometric occlusion detection (computation): apply the occlusion-aware point-based method to the complete scene.
5. Wave-based occlusion detection (input+computation): apply the silhouette-mask layer-based method to the rasterized scene.
6. Wave-based occlusion detection (computation): apply the silhouette-mask layer-based method to the complete scene.

Figure 9 visualizes the result, and we conclude four types of artifacts:

1. Leakage of the wavefront from occluded points.
2. Leakage of the wavefront from visible points.
3. Loss of wavefront from occluded points.
4. Inaccurate wavefront due to geometric optics modeling.

Modeling occlusion at the scene representation stage (setting 2) avoids the type 1 artifact and reduces the input data size. Applying either type of occlusion detection at the computation stage (setting 3 or 5) removes the type 2 artifact. If occlusion detection is wave-based (setting 5), the type 4 artifact is also removed, resulting in an artifact-free foreground reproduction. However, the type 3 artifact persists (for setting 2, 3, and 5) due to the loss of occluded points and their wavefronts, causing loss of amplitude or attenuation at the background side of the occlusion boundaries, which are the dominating artifact in TensorHolo V1. Retaining the complete scene and applying wave-based occlusion detection (setting 6) avoid all types of artifacts with the defocus response of the background bars matching the ones in setting 1. However, this mode incurs a higher data and computational cost.

#### Algorithm 1 Depth peeling for LDI rendering


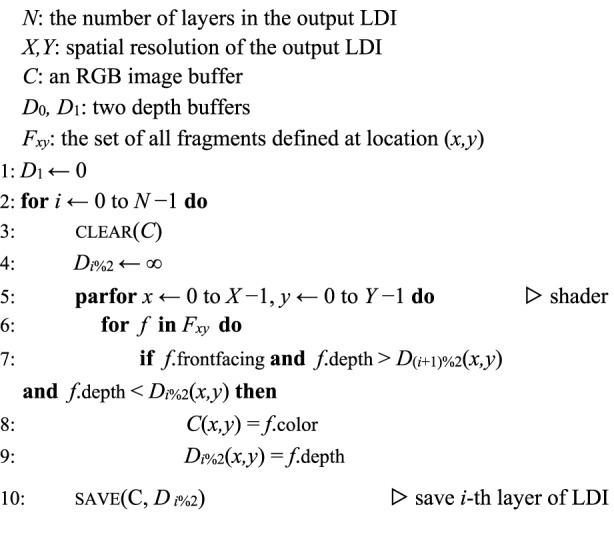


### Depth peeling for LDI rendering

Depth peeling is a rendering method originally developed for order-independent transparency (https://developer.download.nvidia.com/assets/gamedev/docs/OrderIndependentTransparency.pdf), and an LDI is an intermediate product of the algorithm. To generate a *N*-layer LDI, depth peeling runs the rendering pipeline *N* times and simultaneously maintains two depth (*Z*) buffers. One works conventionally, while the other remains a constant at each rendering pass and sets the minimum distance at which a fragment can be drawn without being discarded. For each pass, the previous pass’s conventional Z-buffer is used as the current minimal Z-buffer so that the content right behind the previous pass is rendered. Algorithm 1 outlines the pseudocode of depth peeling.

### Details of training and runtime performance of TensorHolo V2

**Fig. 10:**
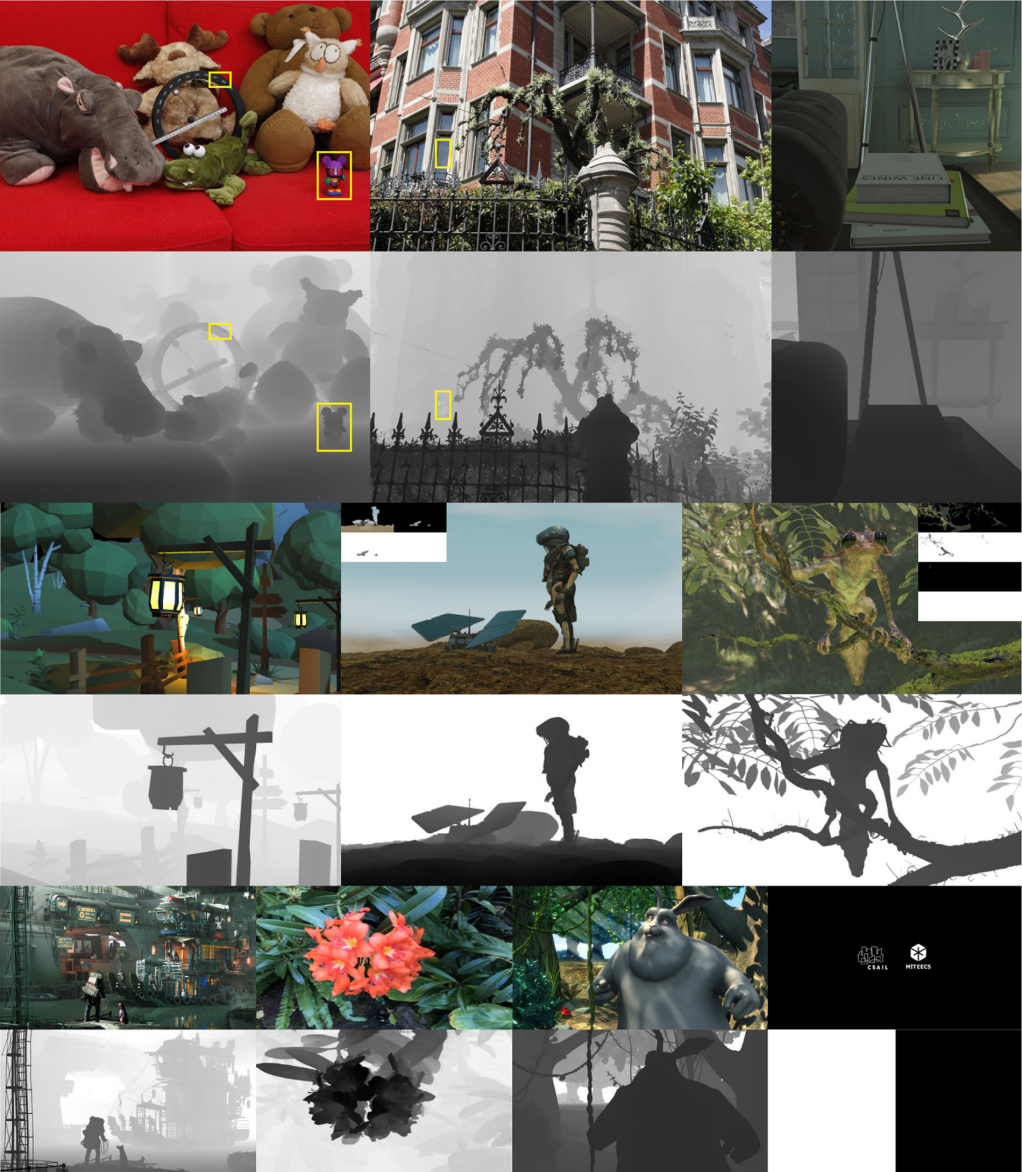
RGB-D and LDI inputs used in this paper. From top to bottom and left to right: ‘Couch’ and ‘Mansion’ from Kim et al.^51^, a living room scene from Xiao et al.^52^, a forest scene from Padmanaban et al.^32^, ‘Wanderer’ and ‘Tree Creature’ by Daniel Bystedt, ‘PartyTug 6:00AM’ by Ian Hubert, ‘Flower’ from Mildenhall et al.^53^, ‘Big Buck Bunny’ by (© 2008, Blender Foundation), and ‘MIT-EECS-Logo’ by the authors. ‘Couch’, ‘Mansion’, and ‘Flower’ are real-world captured, and the rest are rendered. ‘Wanderer’ and ‘Tree Creature’ are LDIs and the subsequent layers are visualized at the top corners. The yellow boxes mark the regions with obvious depth misalignment or inconsistency, the soft leave edges in the depth map of’Flower’ are not marked

The CNNs are implemented and trained using TensorFlow 1.15 on an NVIDIA RTX 8000 GPU with Adam optimizer. The hologram synthesis CNN uses the same residual network architecture as the V1 CNN, which consists of 30 convolution layers with 24 3 × 3 kernels per layer (see ref. ^6^ for the discussion of design choice). The pre-filtering CNN uses the same architecture but with only eight convolution layers and 8 3 × 3 kernels per layer. The pre-filtering CNN can be omitted when the target hologram coincides with the midpoint hologram. The learning rate is 0.0001 with an exponential decay rate of *β*_1_ = 0.9 for the first moment and *β*_1_ = 0.99 for the second moment. The first stage training runs for 3000 epochs. The second stage training first pre-trains the pre-filtering CNN 50 epochs for identity mapping and then 1000 epochs jointly with the hologram synthesis CNN. The pre-training accelerates the convergence and yields better results. Both versions of CNN use a batch size of 2, *w*_data_ = 1.0, *w*_pcp_ = 1.0, *w*_tgt-pre_ = 0.07, where *w*_data_, *w*_pcp_, *w*_tgt-pre_ are the weights for the data fidelity loss, the dynamic focal stack loss, and the regularization loss. Other parameters remain the same as TensorHolo V1. Table 3 lists the runtime performance of TensorHolo V2 RGB-D CNNs on an NVIDIA TITAN RTX GPU and an iPhone 13 Pro. The rendered LDIs and real-world captured RGB-D inputs can be found in Fig. 10. In particular, the speed improvement on the mobile device is following Moore’s law (1.1 to 5 Hz from iPhone 11 Pro to 13 Pro), which could promise real-time performance in a horizon of 5 years if this trend continues. Other dedicated ASICs for CNN, such as Graphcore IPU, Google TPU, and Groq TSP, may also enable efficient edge and cloud hologram computation and streaming for personal entertainment and enterprise devices.

**Table 3:** Runtime performance of TensorHolo V2 RGB-D CNNs on GPU and iPhone 13 Pro

	30 layers	15 layers	8 layers
NVIDIA TITAN RTX	40 ms	25 ms	17 ms
iPhone 13 Pro	478 ms	297 ms	209 ms

The numbers are reported for the CGH synthesis networks with a different number of convolution layers. When the target hologram plane offsets from the midpoint hologram, the addition of the pre-filtering CNN adds 4.8 ms for a TITAN RTX and 60 ms for an iPhone 13 Pro.

### Details of the experimental setup

The setup (see Fig. 6) uses a HOLOEYE PLUTO (VIS-014) phase-only LCoS with a 1920 × 1080 pixels resolution and a pixel pitch of 8 um. This SLM provides a refresh rate of 60 Hz (monochrome) with a bit depth of 8 bits. The laser is a FISBA RGBeam single-mode fiber-coupled module with three optically aligned laser diodes at 638, 520, and 450 nm wavelengths. The diverging beam emitted by the laser is collimated by a 300 mm achromatic doublet (Thorlabs AC254-300-A-ML) and polarized (Thorlabs LPVISE100-A) to match the SLM’s function polarization direction. The beam is directed to the SLM by a beamsplitter (Thorlabs BSW10R), and the SLM is mounted on a linear translation stage (Thorlabs XRN25P/M). When displaying holograms with different relative positions to the 3D volumes, we adjust the linear translation stage to keep the position of 3D volumes stationary and thus avoid modifying the following imaging optics. The modulated wavefront is imaged by a 125 mm achromat (Thorlabs AC254-125-A-ML) and magnified by a Meade Series 5000 21 mm MWA eyepiece. An aperture is placed at the Fourier plane to block excessive light diffracted by the grating structure and higher-order diffractions. A SONY A7M3 mirrorless full-frame camera paired with a 16−35 mm f/2.8 GM lens is used to photograph the results. A Labjack U3 USB DAQ is used to send field sequential signals and synchronize the display of color-matched phase-only holograms.

### Compensating hardware imperfection

Hardware imperfection can cause experimental results to deviate from the idealized simulations^23,24^. Here we discuss methods to compensate for three sources of error: laser source intensity variation as a Gaussian beam, SLM’s non-linear voltage-to-phase response, and optical aberrations.

To calibrate the laser source intensity variation, we substitute the SLM with a diffuser and capture the reflected beam as a scaling map for adjusting the target amplitude. A 5 × 5 median filter is applied to the measurements to avoid pepper noise caused by dust on the optical elements. A Gaussian mixture model can be used to fit an analytical model of the resulting scaling map if needed^24^.

**Fig. 11:**
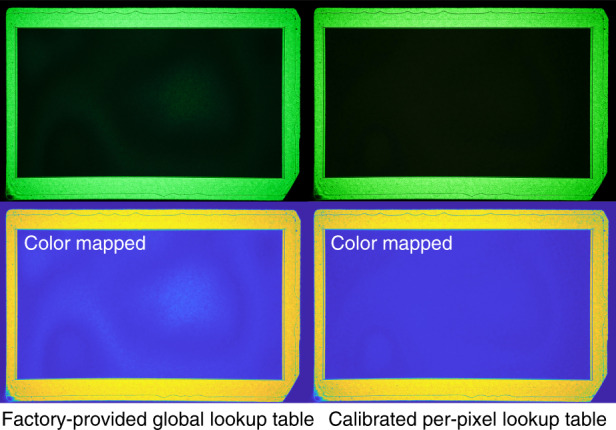
Black modulation test using the double phase encoding. Using the per-pixel lookup table calibrated via the proposed approach produces a more uniform image with a deeper black level over the one achieved by the factory-provided global lookup table.

For an imprecisely calibrated SLM, the non-linear voltage-to-phase response can severally reduce display contrast, especially for double-phase encoded hologram, since achieving deep black requires offsetting the checkerboard grating accurately by 1*π*. In many cases, the pixel response is also spatially non-uniform; thus, using a global look-up table is often inadequate (see Fig. 11). Existing calibration methods operate on the change of interference fringe offset (interferometry-based) or the change of near/far-field diffraction pattern (diffraction-based), but they cannot produce a per-pixel look-up table (LUT)^50^. We propose a simple calibration procedure that uses double phase encoding to accomplish this goal. Specifically, for every 2-by-2 pixels, we keep the top right and bottom left pixels at 0 as a reference and increase the top left and bottom right pixels jointly from 0 to 255. Without modifying the display layout, we set the camera focus on the SLM and capture the intensity change for the entire frame. If the phase modulation range for the operating wavelength is greater equal than 2*π*, the intensity of the captured image will decrease to the minimum at 1*π* offset, return to the maximum at 2*π* offset, and repeat this pattern for every 2*π* cycle. Denote the *k*th captured image *I*_*k*_, the absolute angular difference in the polar coordinate between a reference pixel and an active pixel set to *k* is

12 $$\theta _k(x,y) = 2{{{\mathrm{cos}}}}^{ - 1}\left( {\frac{{\sqrt {I_k} (x,y) - \sqrt {I_{{{{\mathrm{min}}}}}} (x,y)}}{{\sqrt {I_{{{{\mathrm{max}}}}}} (x,y) - \sqrt {I_{{{{\mathrm{min}}}}}} (x,y)}}} \right)$$

where *I*_min_(*x*, *y*) and *I*_max_(*x*, *y*) are the minimal and maximal intensities measured at a location (*x*, *y*) when sweeping from 0 to 255. Let *k*_min_(*x*, *y*) be the frame id associated with the minimal measurement at (*x*, *y*), the phase difference is given by

13 $$\phi _k\left( {x,y} \right) = \left\{ {\begin{array}{*{20}{l}} {\theta _k(x,\,y),\;\;k \le k_{min}(x,\,y)} \\ {2\pi - \theta _k(x,\,y),\;\;k \,>\, k_{min}(x,\,y)} \end{array}} \right.$$

Experimentally, we take high-resolution measurements (24 megapixels) of the SLM response, downsample to the SLM resolution, perform the calculations above, and fit a linear generalized additive model (GAM) with monotonic increasing constraint to obtain a smoothed phase curve for producing a per-pixel LUT. For simplicity, the LUT is directly loaded into the GPU memory for fast inference. To reduce memory consumption, a multi-layer perceptron can be learned and applied as a 1 × 1 convolution^24^. This in-situ calibration procedure eliminates potential model mismatch between a separate calibration setup and the display setup. The ability to accurately address phase differences results in more accurate color reproduction, i.e., producing deep black by accurately addressing 1*π* phase offset (see Fig. 11).

The optical aberrations are corrected using a variant of Maimone et al.^14^. Let $\phi ^\prime _d \in {\Bbb C}^{R_x \times R_y}$ (zero-padded to the frame resolution) be an ideal sub-hologram that focus plane wave to a signed distance *d;* we similarly use 5 Zernike polynomials:

14 $$Z_3\left( {\rho ,\theta } \right) = a_{3_d}\left( {2\rho ^2 - 1} \right)\quad\qquad{{{\mathrm{focus}}}}$$

15 $$Z_4\left( {\rho ,\theta } \right) = a_{4_d}\left( {\rho ^2\cos 2\theta } \right)\quad\qquad{{{\mathrm{vertical}}}}\;{{{\mathrm{astigmatism}}}}$$

16 $$Z_5\left( {\rho ,\theta } \right) = a_{5_d}\left( {\rho ^2\sin 2\theta } \right)\quad\qquad{{{\mathrm{oblique}}}}\;{{{\mathrm{astigmatism}}}}$$

17 $$Z_6\left( {\rho ,\theta } \right) = a_{6_d}\left( {\left( {3\rho ^2 - 2} \right)\rho \cos \theta } \right)\quad\qquad{{{\mathrm{horizontal}}}}\;{{{\mathrm{coma}}}}$$

18 $$Z_7\left( {\rho ,\theta } \right) = a_{7_d}\left( {\left( {3\rho ^2 - 2} \right)\rho \sin \theta } \right)\quad\qquad{{{\mathrm{vertical}}}}\;{{{\mathrm{coma}}}}$$

19 $$\phi _d\left( {\rho ,\theta } \right) = \phi _d^\prime \left( {\rho ,\theta } \right){\rm{e}}^{i\mathop {\sum }\limits_j \left( {Z_j\left( {\rho ,\theta } \right)} \right)}\quad\qquad{{{\mathrm{corrected}}}}\;{{{\mathrm{sub - hologram}}}}$$

to model system aberrations, where $a_{j_{d}}$ are Zernike coefficients, *ρ* is the normalized polar radius, and *θ* is the azimuthal angle. We perform a user calibration to adjust coefficients *a*_*jd*_ until the camera images a tightly focused spot at *d* from the corrected sub-hologram *ϕ*_*d*_. Once the calibration completes, we propagate *ϕ*_*d*_ to its focal plane to obtain the point spread function and compute the corrected amplitude transfer function as $\Phi _d = {{{\mathrm{ATF}}}}_d = {{{\mathcal{F}}}}({{{\mathrm{PSF}}}}_d) = {{{\mathcal{F}}}}({{{\mathrm{ASM}}}}(\phi _d,d))$, which we use in Eq. (5) to perform frequency-domain aberration correction for the occlusion-processed layer. Note that this calibration procedure can be performed for different focal distances, and parameters can be piecewise linearly interpolated^14^.

For compact eyeglass-like setups (i.e., Maimone et al.’s compact AR prototype), the same procedure can be followed by determining the unmagnified image location and the optical power of the diffractive lens while calibrating the kernel parameters to correct the system and eye aberrations jointly. Using a diffractive lens can yet cause strong aberrations that require spatially-varying aberration correction. In this case, we can calibrate the display at multiple points (i.e., 15 points) and update the above procedure by convolving a spatially varying PSF_*d*_(*x*, *y*) calculated by interpolating the nearest measured parameters. Note that this operation can only be performed in the spatial domain but not in the Fourier domain. However, GPUs can accelerate this process, and speed is ultimately not critical for dataset generation. On the learning side, the CNN needs to receive an additional two-channel image that records the normalized *xy* coordinates to learn aberration correction in a spatially-varying manner. While this advanced task is exciting with a clear solution path, we defer it to future work.

## Supplementary information

Supplementary Video 1

Supplementary Video 2

Supplementary Video 3

Supplementary Video 4

Supplementary Information

## Data Availability

The MIT-CGH-4K-V2 dataset, the pre-trained CNN models, and the code to evaluate/train the CNN models will be made publicly available on GitHub
