# Supplementary material for: End-to-end learning of 3D phase-only holograms for holographic display
Source: Light Sci Appl. 2022 Aug 3;11:247. doi: 10.1038/s41377-022-00894-6 (PMC9349218; doi:10.1038/s41377-022-00894-6)
Supplement: Supplementary file 5 — Supplementary Information [file 41377_2022_894_MOESM5_ESM.docx]

Supplementary file details for

**End-to-end learning of 3D Phase-only Holograms for Holographic Display**

Liang Shi, Beichen Li, Wojciech Matusik

Computer Science and Artificial Intelligence Laboratory,

Massachusetts Institute of Technology,

32 Vassar St, Cambridge, MA, 02139, USA

**Supplementary Video 1**

The Focal sweep of a simulated 3D Big Buck Bunny Scene. This video provides visualization of focal sweeps reconstructed using the complex hologram predicted by the TensorHolo V2-RGBD network trained after the 1^st^ stage training and using the phase-only hologram predicted by the TensorHolo V2-RGBD network trained after the 2^nd^ stage training. The phase-only hologram is predicted for the 3D volume with its center offset 12mm away from the physical SLM to show the robustness of the proposed algorithm. The input RGB-D image is computer rendered.

**Supplementary Video 2**

The Focal sweep of a simulated 3D couch scene. This video provides visualization of focal sweeps reconstructed using the complex hologram predicted by the TensorHolo V2-RGBD network trained after the 1^st^ stage training, and the phase-only hologram predicted by the TensorHolo V2-RGBD network trained after the 2^nd^ stage training. The phase-only hologram is predicted for the 3D volume with its center offset 12mm away from the physical SLM to show the robustness of the proposed algorithm. The input RGB-D is from the ETH Light Field dataset, where the RGB image is physically captured by a Canon EOS 5D Mark II DSLR camera, and the depth is estimated by the algorithm of [Kim et al. 2013].

[Kim et al. 2013]. 1. C. Kim, H. Zimmer, Y. Pritch, A. Sorkine-Hornung, M. Gross, Scene reconstruction from high spatio-angular resolution light fields. *ACM Trans. Graph.* **32**, 1–12 (2013).

**Supplementary Video 3**

This video demonstrates a photographed focal sweep of a TensorHolo V2-RGBD predicted Big Buck Bunny 3D phase-only hologram. The video is captured by a Sony A7 Mark III mirrorless camera paired with a Sony GM 16-35mm/f2.8 camera lens. Only the green channel is visualized for temporal stability.

**Supplementary Video 4**

This video demonstrates real-time 3D hologram computation on an NVIDIA TITAN RTX GPU. The CGH synthesis CNN has a reduced capacity of 8 layers and 30 filters/layer to enable 60 frames per second runtime speed. The input RGBD sequence is modified from the results of NeRF [Mildenhall et al. 2020]. The video is captured by a Panasonic GH5 mirrorless camera with a Lumix 10-25 mm f/1.7 lens. The full-color reconstruction is obtained field sequentially. The inactive border of the SLM is intentionally included to show the intensity of the illumination and the contrast of the display.

[Mildenhall et al. 2020] B. Mildenhall, P. P. Srinivasan, M. Tancik, J. T. Barron, R. Ramamoorthi, R. Ng, in *Computer Vision – ECCV 2020* (Springer International Publishing, 2020), pp. 405–421.
